# Supplementary material for: Characterization of the Cynomolgus Macaque Model of Marburg Virus Disease and Assessment of Timing for Therapeutic Treatment Testing
Source: Viruses. 2023 Nov 28;15(12):2335. doi: 10.3390/v15122335 (PMC10748006; doi:10.3390/v15122335)
Supplement: Supplementary file 1 [file viruses-15-02335-s001.zip › viruses-2721828-supplementary.pdf]

viruses

MDPI

Supplemental Material

Supplemental Methods

Animal Inclusion and Exclusion Criteria and Health Screens

Animal health evaluations prior to study start included a physical exam, behavioral assessment, and evaluation of clinical pathology parameters. In addition, animals were subjected to health screens within 6 months prior to the initiation of jacket acclimation to ensure that they met the following inclusion criteria: negative retroviral screening tests (simian-T-lymphotropic virus-1; simian immunodeficiency virus; and simian retrovirus-1, -2, and -3), negative polymerase chain reaction (PCR) and immunoglobulin G (IgG) screening tests for *Trypanosoma cruzi*, negative herpes B test, the final of three consecutive negative tuberculosis tests, and stool cultures negative for Salmonella and Shigella. Exclusion criteria were used to ensure that none of the animals were under treatment for an existing disease condition or injury within 30 days prior to study initiation, had a history of gastrointestinal disorders requiring treatment within 30 days prior to study initiation, or had preexisting antibodies (IgG) to Marburg virus (MARV) glycoprotein.

Animal Husbandry

Animals were housed in accordance with the US Department of Agriculture Animal Welfare Act (9 Code of Federal Regulations [CFR], Parts 1, 2, and 3), and the Guide for the Care and Use of Laboratory Animals.[45] Animals were maintained on a 12-hour light/12-hour dark photoperiod, except when room lights were turned on for study-related procedures (e.g., animal observations). Temperatures were targeted between 64°F and 84°F (18°C–29°C) with a relative humidity of 30–70%.

2050C Certified Monkey Diet (Envigo Teklad), was provided daily to animals. Additional dietary enrichment, such as fruits, were also provided. For each lot of 2050C feed, the manufacturer analyzes maximum allowable concentrations of contaminants (e.g., heavy metals, aflatoxin, organophosphates, chlorinated hydrocarbons, and polychlorinated biphenyls). The specifications for acceptable levels of contaminants were the established maximum concentrations as they appear on the certificate of analysis of the feed used during the study. Each lot of 2050C feed is tested for microbial and chemical toxicants. Water was provided *ad libitum* via an automatic watering system or water bottles. Oral rehydration solution (Pedialyte mixed with water) was provided to all animals in Groups 1 and 2 from the day of exposure onward. The volume of Pedialyte provided to each animal was evaluated once daily and the remaining percentage volume was documented in study records.

Surgical Procedures

Two surgical procedures were conducted 20–23 days prior to challenge and after at least 6 days of acclimation to the Lomir primate jacket and tethering system. To acquire body temperature and activity data, a DSI M00 telemetry device was surgically implanted into each animal. The transmitter was implanted in a pocket created underneath the external abdominal oblique muscle. To allow for venous access, a central venous catheter (CVC) was surgically implanted into each animal at the same time as the telemetry implantation. During surgery, anesthesia was maintained and intra-operative thermal support, fluid support, analgesic (bupivacaine), and antibiotic were provided.. After surgery, correct placement of the catheter was confirmed by radiography and each animal was placed in a primate jacket. The nonhuman primates (NHPs) were extubated after the swallow reflex returned. Animals were observed for

complications following surgery. Animals were allowed to recover from surgery for at least 11 days before transfer to the biosafety level 4 (BSL-4) facility in which the study was conducted.

Necropsy

Necropsies on all animals were conducted by a board-certified veterinary pathologist in the BSL-4 suite. The necropsy included examination of the carcass and musculoskeletal system; all external surfaces and orifices; the neck with associated organs and tissues; and cranial, thoracic, abdominal, and pelvic cavities with their associated organs and tissues. Tissues examined and collected included: lung, aorta, esophagus, trachea, heart, liver, spleen, kidney, urinary bladder, skin (w/rash if present), inoculation site, adrenal gland, lymph nodes (axillary, inguinal, mesenteric, and tracheobronchial), stomach, pancreas, small intestine, large intestine, brain, eye, skeletal muscle, sciatic nerve, uterus, ovary, prostate gland, testes, and epididymis. Tissues and organs were first examined *in situ*, then were dissected from the carcass. Specimens were collected and fixed by immersion in labeled containers of 10% neutral buffered formalin. All gross findings were recorded per individual animal in descriptive terms, including location(s), size, shape, color, consistency, and number as appropriate. All tissues with significant gross lesions were also collected and recorded. All necropsy and tissue collection data were maintained in Pristima (version 7.4.0, build 16).

After a minimum of 21 days of formalin fixation, each specimen container was removed from the BSL-4 suite. Tissues were transported to the histology laboratory. The tissue samples were trimmed, routinely processed, and embedded in paraffin. The paraffin-embedded tissues were cut for histology in 5-µm sections. After the paraffin-embedded tissues were cut and placed on glass slides, they were deparaffinized, stained with hematoxylin and eosin, and coverslipped. Replicate tissue sections were placed on positively charged slides and stained for immunohistochemistry (IHC) using a mouse monoclonal antibody (USAMRIID #704). To detect MARV genomic RNA in formalin-fixed, paraffin-embedded tissues, *in situ* hybridization (ISH) was performed using the RNAscope 2.5 HD RED kit according to the manufacturer's instructions and USAMRIID standard operating procedures. Briefly, ISH probes targeting the genomic MARV nucleoprotein gene were designed and synthesized by Advanced Cell Diagnostics. Tissue sections were deparaffinized and were exposed to ISH target probe pairs. ISH signal was amplified using kit-provided Pre-amplifier and Amplifier conjugated to alkaline phosphatase and incubated with a Fast Red substrate solution for 10 minutes at room temperature. Sections were then stained with hematoxylin, air-dried, and mounted.

Statistical Analysis

The natural disease progression and clinical changes resulting from intramuscular (IM) injection of MARV in cynomolgus macaques were evaluated through analysis of the following endpoints: overall survival, MARV viral load assessments (plasma MARV RNA and infectious virus plaque serum viremia), clinical laboratory parameters (coagulation, hematology, and chemistry), body weight, telemetry assessments (body temperature and activity levels), and disease states and their relation to clinical laboratory parameters. The SAS programs contain the SAS code necessary to generate the analysis. The SAS Log files are the log of the SAS programs as run for the final dataset and creation of tables, figures, and listings. The SAS version number was SAS 9.4.

Animal demographic data and baseline characteristics were compared between exposure groups using Fisher's exact tests (for categorical data) and Wilcoxon rank-sum tests (for continuous data). The overall survival rate of MARV-exposed vs. mock-exposed animals was compared using a Fisher's exact test. Animals that did not reach euthanasia criteria or death but were euthanized according to protocol pre-specified time were considered to have survived for analysis. Survival curves were constructed using the Kaplan–Meier method, and curves were compared between

MARV-exposed and mock-exposed animals by log-rank test. Mock-exposed animals were censored on the date on which the final MARV-exposed animal succumbed or was euthanized.

To assess the relationship between MARV exposure and viral load, the following endpoints were explored and summarized using descriptive statistics: MARV RNA (log_{10} ge/mL) values by study day, last available MARV RNA (log_{10} ge/mL) value, maximum MARV RNA (log_{10} ge/mL) value, time-weighted average MARV RNA value, and infectious virus in serum (log_{10} ge/mL) values by study day.

For analyses and summaries shown in Tables S5 and S6, the Wilcoxon matched pairs test was used to compare the MARV dataset on each day to the MARV baseline. In addition, the Mann–Whitney U test was used to compare the MARV dataset on each day to the mock dataset on the same day. These two tests were conducted using GraphPad Prism 9.

All laboratory data collected on or after the date of infection up to the end of the study were summarized by exposure group using descriptive statistics. For hematology, coagulation, and chemistry parameters, MARV- and mock-exposed animals were compared using the Wilcoxon rank-sum test for the value at each study day and the change from the day of infection (Day 0) to each Day post inoculation (PI). Comparisons of the changes from the day of infection (Day 0) within an exposure group were made using the Wilcoxon signed rank test for each study day.

Daily maximum animal responsiveness scores were summarized at each study day by exposure group.

Body weight data were summarized using descriptive statistics by exposure group for the day of infection (Day 0) and the terminal day (time of euthanasia or time of necropsy for animals found deceased).

Time-matched body temperature and activity levels measured via telemetry at baseline, post-baseline, and change from baseline were summarized by study day using descriptive statistics by exposure group. Comparisons of MARV-exposed and mock-exposed animals' temperature and activity data were made using the Wilcoxon rank-sum test for the time-matched value and time-matched change from baseline for each study day. Comparisons of the time-matched changes from baseline within an exposure group were made using the Wilcoxon signed rank test for each study day.

The time (in hours) from MARV or mock exposure (Day 0) to several telemetry-based and animal responsiveness score-based disease states were summarized using descriptive statistics. Time between the key disease states of interest were summarized similarly for MARV-exposed animals only. The percentage of animals reaching each disease state was compared between MARV-exposed and mock-exposed animals using Fisher's exact test.

Supplemental Results

Cage-Side Observations

Rash

None of the mock-exposed animals had rash at any time point. Conversely, 10 of 12 MARV-exposed animals were observed to have a rash at some point over the disease course during cage-side observations (Table S1). Mild/localized rash was first observed in 3 of 12 animals on Day 6 PI. On Day 7 PI, rash was observed in 8 of 12 animals, with 3 recorded as mild (light/barely visible) and 5 moderate (easily visible/distinct), and ranged in distribution from localized (isolated) in 4 animals to limited (present in select regions) in 4 animals. By Day 8 PI, 9 of 11 animals had a rash, with 1 animal having a severe rash (distinct, very dark color). At Day 9 PI, all 3 remaining MARV-exposed animals had a rash, ranging from mild to severe and with a localized to widespread distribution. The rash observed in MARV-exposed animals did not resolve after onset in any of the 10 animals for which rash was visible during cage-side observations.

These rash findings, from cage-side observations, differ slightly from those observed during the final anesthetized observations, as it was easier to see a rash during the anesthetized exam during which the catheter jacket is removed. Notably, a rash was not observed during cage-side observations of NHPs 9 and 13, but during the anesthetized physical exam just prior to euthanasia, both of these animals had mild rash with limited distribution. Thus, under anesthesia, it was apparent that all 12 of 12 MARV-exposed animals exhibited a rash, which is attributed to MARV exposure.

Urine and Stool

Urine appeared normal in both mock-exposed and MARV-exposed animals throughout the study. Stool was nearly always present and appeared normal in all mock-exposed animals throughout the study. Most MARV-exposed animals developed some abnormality to the stool (Table S1). Specifically, 11 of 12 animals had no stool observed on one or more days, starting on Day 5 PI, with stool absent from the cages of 3 of 12 NHPs. By Day 7 PI, 10 of 12 animals did not have stool observed in the cage. Additionally, 1 NHP, animal 11, had loose stool on Day 4 PI. No other stool abnormalities were observed.

Food Intake (Biscuits and Fruit)

All mock-exposed animals had evidence of biscuit consumption on each day of the study. In contrast, all 12 MARV-exposed animals had evidence of reduced biscuit consumption at some point in the disease course, beginning on Day 5 PI for 4 of 12 animals (Table S1). On Day 6 PI, the majority of MARV-exposed NHPs (9 of 12) did not show evidence of biscuit consumption and, on Day 7 PI, none of the MARV-exposed animals ate any of their daily allotment of biscuits. This trend continued through the end of the study with 7 of 8 and 3 of 3 MARV-exposed animals showing no evidence of biscuit consumption on Days 8 and 9 PI, respectively.

Animals also received daily food enrichment, such as fresh fruit. All mock-exposed animals consumed the enrichment on each day of the study. MARV-exposed animals also generally consumed the food enrichment. However, 4 of 12 MARV-exposed animals did not consume the enrichment on Day 6 PI, and 2 of 8 animals did not consume enrichment on Day 8 PI (Table S1).

Table S1. Summary of Cage-Side Observations in MARV-Exposed NHPs

Observation	Number of Animals Affected on Each Day PI						
	Day 4 PI	Day 5 PI	Day 6 PI	Day 7 PI	Day 8 PI	Day 9 PI	Day 10 PI
Impaired Motor Function	0 of 12	4 of 12	5 of 12	8 of 12	9 of 11	3 of 3	1 of 1
Rash	0 of 12	0 of 12	3 of 12	8 of 12	9 of 11	3 of 3	1 of 1
Bleeding	1 of 12	0 of 12	0 of 12	0 of 12	1 of 11	0 of 3	0 of 1
Facial Swelling	0 of 12	0 of 12	0 of 12	1 of 12	0 of 11	0 of 3	0 of 1
Loose Stool	1 of 12	0 of 12	0 of 12	0 of 12	0 of 8	0 of 3	NA
Vomit	0 of 12	1 of 12	2 of 12	0 of 12	0 of 11	0 of 3	0 of 1
No Biscuits Consumed	0 of 12	4 of 12	9 of 12	12 of 12	7 of 8	3 of 3	NA
No Enrichment Consumed	0 of 12	0 of 12	4 of 12	0 of 12	2 of 8	0 of 3	NA
Decrease in Pedialyte[a]	3 of 12	1 of 12	8 of 12	10 of 12	12 of 12	4 of 4	1 of 1
No Stool	0 of 12	3 of 12	8 of 12	10 of 12	7 of 8	3 of 3	NA

NA, not applicable. Orange shading corresponds with the proportion of animals affected by each change, with darker tints indicating a greater proportion of animals affected. The total number of animals included each day is the number for which a given parameter could be analyzed; as the study progressed, some parameters could not be assessed due to worsening animal condition and to animals succumbing. [a] Decrease in consumption of Pedialyte is defined as having 75–100% Pedialyte remaining in the bottle. Note that 3 animals (10, 16, and 18) had 75–100% Pedialyte remaining on at least 2 days from Day 1 through Day 3 PI.

Pedialyte

Freshly prepared Pedialyte was provided daily to each animal and any remaining volume was qualitatively assessed and documented the following day (as "<25%," "25–49%," "50–74%," or "75–100%"), along with observations on the status of whether the Pedialyte bottle remained assembled and attached to the cage.

Changes in remaining Pedialyte volume, while suggestive of consumption, do not definitively indicate that all or any of the Pedialyte was consumed. Volume changes can result from leakage or from physical contact between the animal and the valve. As a result, findings regarding Pedialyte volumes should be interpreted with caution.

In general, 5 of 6 mock-exposed animals consumed Pedialyte on most days of the in-life portion of the study. One exception was animal 4, which showed decreased Pedialyte consumption on Days 6–10 PI. Of the MARV-exposed animals, 3 NHPs (animals 10, 16, and 18) consistently did not drink Pedialyte, from the day of challenge onward. The remaining 9 MARV-exposed animals demonstrated more consistent consumption of Pedialyte on Days 0–4 PI. On Day 5 PI, 1 of 12 NHPs showed decreased Pedialyte consumption and, by Day 7 PI, 10 of 12 NHPs demonstrated decreased Pedialyte consumption (Table S1).

Motor Function

All mock-exposed animals retained normal motor function throughout the study per cage-side observations. All MARV-exposed animals (12 of 12) exhibited mild to severe motor dysfunction, first observed in 4 of 12 animals on Day 5 PI (Table S1). The number of animals with impaired motor function increased as the study progressed, with 5 of 12 (Day 6 PI), 8 of 12 (Day 7 PI), 9 of 11 (Day 8 PI), 3 of 3 (Day 9 PI), and 1 of 1 (Day 10 PI) impacted. The motor dysfunction was mild or mild to moderate in most animals. However, 3 MARV-exposed NHPs (animals 10, 12, and 13) developed severe motor dysfunction. Additionally, 7 NHPs were described as "comatose" at the awake observation immediately preceding euthanasia (animals 8, 9, 14, 15, 18, 13, and 17).

Infrequent Observations

Bleeding was not observed in any mock-exposed animals with one exception. On Day 7 PI, animal 3 was observed to have facial bleeding from the left nostril, and the technician performing the assessment stated that this was likely due to "aggressive biting of toy, as witnessed during observation." In MARV-exposed animals, instances of bleeding were observed in 2 of 12 NHPs during cage-side observations. Animal 14 had blood under both nares on Day 4 PI. Animal 18 had a mixture of fresh and clotted blood in its mouth and from its nose on Day 8 PI. These cage-side observations of bleeding differ slightly from those observed during the final anesthetized observation, as it was easier to see the bleeding during the anesthetized observations. Notably, bleeding was not observed during cage-side observations of animals 9 and 12, but during the anesthetized physical exam just prior to euthanasia, both of these animals had mild bleeding (vaginal and mouth, respectively). Thus, a total of 4 MARV-exposed animals exhibited bleeding.

Facial swelling was not observed in any mock-exposed animals on any day of the study. In the MARV-exposed group, 1 of 12 NHPs (animal 13) had facial swelling at three observations on Day 7 PI.

Evidence of vomiting was observed in 2 of 12 animals in the MARV-exposed group. Vomit was found on the bottom of the cage of animal 13 on Day 6 PI. Vomit was also found on the bottom of the cage of animal 17 on Day 5 PI; on Day 6 PI, this animal vomited during a cage-side observation. No mock-exposed animals had any evidence of vomiting during this study.

Euthanasia Narrative

Upon assignment of a responsiveness score of 4, and prior to anesthetizing the animal to perform euthanasia, a euthanasia narrative was completed cage-side to document the clinical attributes of the animal at the time the responsiveness score was assigned. Findings from these observation events are summarized in Table S2. Nine of 12 MARV-exposed NHPs received a score of 4 and a euthanasia narrative was completed; 3 of 12 NHPs were found deceased and, therefore, a euthanasia narrative was not completed. When a responsiveness score of 4 was assigned, MARV-exposed nonsurvivors exhibited alterations in physical appearance, skin color, position, posture, responsiveness to observers, responsiveness to other NHPs in the room, and behavior. Six of 9 animals had an abnormal physical appearance, with dull or ungroomed fur. All 9 MARV-exposed animals receiving a responsiveness score of 4 had abnormal skin color, with 7 of 9 described as pale, 2 of 9 with a yellowish tint, and 1 of 9 with a bluish tint (these categories are not mutually exclusive; animal 18 was described as both pale and having a yellowish tint). Most (8 of 9) MARV-exposed NHPs were lying down in the cage; however, 1 NHP (animal 9) was sitting, curled tightly on the perch. Overall, 8 of 9 animals were comatose and 1 animal (animal 15) was semi-comatose, only briefly acknowledging the observer. MARV-exposed animals (9 of 9) were not responsive to the observer or other NHPs in the room when the responsiveness score of 4 was assigned.

Table S2. Euthanasia Narrative Summary

**Observations**		**Number of Animals Affected**
**Physical Appearance**	Normal Coat	3 of 9
	Dull	3 of 9
	Ungroomed Fur	3 of 9
**Skin Color**	Pale	7 of 9[a]
	Yellowish Tint	2 of 9
	Blueish Tint	1 of 9
**Position/Posture**	Lying Down	8 of 9
	Sitting on Perch/Hunched	1 of 9
**Responsiveness to Observers**	Not Responding	9 of 9
**Responsiveness to NHPs**	Not Responding	9 of 9
**Activity**	Not Moving Around Cage	9 of 9
**Behavior**	Comatose	8 of 9
	Other	1 of 9

Orange shading corresponds with the proportion of animals affected by each change, with darker tints indicating a greater proportion of animals affected. [a] Animal 18 was observed to have both pale and yellowish skin.

Final Anesthetized Observations

During final physical observations, conducted under anesthesia, the following parameters were assessed: weight, rash, bleeding, discharge, swelling, lymphadenopathy, and inoculation site changes. Final physical observations were recorded for all mock-exposed animals prior to scheduled euthanasia at the end of the study. All parameters were normal for all mock-exposed animals (n = 6). Final anesthetized observations were conducted for all MARV-exposed animals that received a responsiveness score of 4 (n = 9), prior to euthanasia. For the 3 MARV-exposed animals that were found deceased in their cages, final anesthetized observations were not conducted.

Body Weight

The average weights of mock-exposed and MARV-exposed animals were not significantly different on the day of challenge ($p = 0.17$), with an average weight of 3.8377 kg for mock-exposed animals (range 3.4912–4.1113) and an average weight of 4.0539 kg for MARV-exposed animals (range 3.4098-4.4746 kg). Mock-exposed and MARV-exposed animals did not differ significantly in weight change ($p = 0.052$) from the time of challenge to the final weight for each animal before euthanasia. All mock-exposed animals (n = 6) gained weight between the day of challenge and the final anesthetized exam, with an average weight gain of 4.50% (range 2.26–7.19%; Table S3). The average weight of MARV-exposed animals also increased between the day of challenge and the final anesthetized exam, with an average weight gain of 1.14% (range -5.32 to 8.01%). However, in the MARV-exposed group, 6 animals had an increase in weight and 3 animals (animals 9, 14, and 15, noted in red in Table S3) lost weight (range -0.03 to -5.32%). A final weight was not collected on the 3 MARV-exposed animals that were found deceased in the cage. The increase in weight for the mock-exposed group was significant ($p = 0.031$), whereas the increase in weight for the MARV-exposed group was not significant ($p = 0.36$).

Table S3. Change in Body Weights

Group	Subject ID	Weight Day 0[a] (kg)	Final Weight (kg)	Change (kg)	% Change
Mock	1	4.1113	4.260[b]	0.1487	3.62
	2	3.7624	3.9953	0.2329	6.19
	3	3.6605	3.7434	0.0829	2.26
	4	3.4912	3.5728	0.0816	2.34
	5	4.0521	4.2714	0.2193	5.41
	6	3.9484	4.2324	0.2840	7.19
MARV	7	4.4746	NA	NA	NA
	8	4.3130	4.4580	0.1450	3.36
	9	3.6690	3.4738	-0.1952	-5.32
	10	4.3382	4.4661	0.1279	2.95
	11	3.4098	NA	NA	NA
	12	3.8370	4.1444	0.3074	8.01
	13	4.436[b]	4.4966	0.0606	1.37
	14	4.0955	3.9958	-0.0997	-2.43
	15	3.9169	3.9158	-0.0011	-0.03
	16	4.2558	NA	NA	NA
	17	3.7140	3.7664	0.0524	1.41
	18	4.1870	4.2264	0.0394	0.94

Red shading highlights weight loss between Day 0 and the final anesthetized exam. NA: weight was not collected on animals found deceased. [a] Day 0 = day of inoculation. [b] Day 0 weight for animal 13 and final weight for animal 1 were recorded only to the thousands place in raw data.

Rash, Bleeding, and Discharge

During the final anesthetized observations conducted at the end of the in-life phase, none of the mock-exposed animals (0 of 6) exhibited rash. In contrast, during the anesthetized observations of MARV-exposed animals, rash was observed in 9 of 9 NHPs (n = 3 mild, n = 5 moderate, and n = 1 severe; Table S4). For most animals, the rash was limited (n = 4) or widespread (n = 4) in distribution, but 1 animal had only a localized rash. Of note, a terminal exam was not conducted for the 3 MARV-exposed animals that were found deceased; therefore, rash was not evaluated after these animals succumbed.

None of the mock-exposed animals exhibited bleeding at the final anesthetized observations (0 of 6). In contrast, bleeding was observed in 3 of 9 MARV-exposed animals during the final anesthetized observations (Table S4) and, for 2 of these NHPs (animals 9 and 12), the bleeding had not been visible during the cage-side observations. The sites and severity of bleeding were mild vaginal (animal 9), mild mouth (animal 12), and moderate nose and mouth (animal 18).

At the final anesthetized observations conducted at the end of the in-life phase, none of the mock-exposed animals exhibited any signs of discharge (0 of 6). Discharge was observed in 2 of 9 MARV-exposed animals at the final anesthetized physical observation (Table S4). In animal 12, mild discharge from the nose and mouth was observed. Additionally, animal 18 had mild discharge from the mouth.

Table S4. Final Anesthetized Observations in MARV-Exposed NHPs

**Observations**		**Number of MARV-Exposed Animals Affected**
**Rash Severity**	Mild	3 of 9
	Moderate	5 of 9
	Severe	1 of 9
**Rash Distribution**	Localized	1 of 9
	Limited	4 of 9
	Widespread	4 of 9
**Bleeding Source**	None	6 of 9
	Vaginal	1 of 9
	Mouth	1 of 9
	Nose and Mouth	1 of 9
**Bleeding Severity**	NA	6 of 9
	Mild	2 of 9
	Moderate	1 of 9
**Discharge Source**	None	7 of 9
	Mouth	1 of 9
	Nose/Mouth	1 of 9
**Discharge Severity**	NA	7 of 9
	Mild	2 of 9
**Swelling Location/Severity**	None/NA	9 of 9
**Palpable Lymph Nodes**	None	7 of 9
	L/R Inguinal	1 of 9
	L/R Mandibular and L Inguinal	1 of 9
**Inoculation Site**	Normal	9 of 9

Orange shading corresponds with the proportion of animals affected by each abnormal observation, with darker tints indicating a greater proportion of animals affected.

Lymphadenopathy

At the final anesthetized observations conducted at the end of the in-life phase, no lymphadenopathy (palpable lymph nodes) was observed in the mock-exposed animals (0 of 6). In the MARV-exposed group, lymphadenopathy was observed in 2 of 9 NHPs (Table S4). Animal 10 from the MARV-exposure group had lymphadenopathy of the left and right inguinal lymph nodes. Additionally, MARV-exposed animal 15 had lymphadenopathy of the left and right mandibular lymph nodes and the left inguinal lymph node.

Inoculation Site

Inoculation site abnormalities were not observed in any animals on the study, with all mock-exposed animals (6 of 6) and MARV-exposed animals (9 of 9) showing normal appearance of the inoculation site at the final anesthetized observations conducted immediately prior to euthanasia (Table S4).

Plasma Viral RNA (RT-PCR)

Table S5. Plasma Viral RNA (log_{10} ge/mL)

Day PI		Mock	MARV
0	Mean (range)	4.20 (4.20–4.20)	4.20 (4.20–4.20) ^{NA,NA}
	n	6	12
3 (AM)	Mean (range)	4.20 (4.20–4.20)	4.48 (4.20–5.90) ^{ns,ns}
	n	6	12
3 (PM)	Mean (range)	4.20 (4.20–4.20)	4.54 (4.20–5.90) ^{ns,ns}
	n	4	10
4 (AM)	Mean (range)	4.20 (4.20–4.20)	5.95 (4.20–7.57) ***,***
	n	6	12
4 (PM)	Mean (range)	4.20 (4.20–4.20)	6.18 (5.90–7.43) **,***
	n	4	10
5	Mean (range)	4.20 (4.20–4.20)	7.68 (6.15–9.57) ***,***
	n	6	12
6	Mean (range)	4.20 (4.20–4.20)	10.14 (8.93–11.31) **,***
	n	4	10
7	Mean (range)	4.20 (4.20–4.20)	10.69 (10.00–11.17) ***,***
	n	6	11
8	Mean (range)	NA	10.78 (10.23–11.13) *,**
	n	0	6
9	Mean (range)	4.20 (4.20–4.20)	10.61 (10.40–10.76) ^{ns,*}
	n	6	3
10	Mean (range)	NA	10.49 (NA) ^{NA,NA}
	n	0	1
12	Mean (range)	4.20 (4.20–4.20)	NA
	n	6	0

For analyses and summaries, samples in which viral RNA was detected at levels < LLOQ (5.90 log_{10} ge/mL) were assigned the LLOQ, and samples in which viral RNA was not detected (< LOD) were assigned a value of 4.20 log_{10} ge/mL. Statistical significance from within- and between-group comparisons, respectively (see Statistical Analysis above), is noted in superscript next to the MARV RNA values. The significance noted is as follows: ns (not significant) = $p > 0.05$, *$p \le 0.05$, **$p \le 0.01$, ***$p \le 0.001$, ****$p \le 0.0001$. NA = not applicable.

Serum Infectious Virus Load (Plaque Assay)

Table S6. Serum Infectious Virus (log_{10} pfu/mL)

Day PI		Mock	MARV
0	Mean (range)	1.70 (1.70–1.70)	1.70 (1.70–1.70) $^{NA, NA}$
	n	6	12
3	Mean (range)	1.70 (1.70–1.70)	1.70 (1.70–1.70) $^{NA, NA}$
	n	6	12
5	Mean (range)	1.70 (1.70–1.70)	6.79 (3.83–9.18) ***,***
	n	6	12
7	Mean (range)	1.70 (1.70–1.70)	9.08 (8.79–9.18) ***,****
	n	6	11
8	Mean (range)	NA	9.12 (8.93–9.18) *,**
	n	0	6
9	Mean (range)	1.70 (1.70–1.70)	9.09 (9.03–9.18) ^{ns,*}
	n	6	3
10	Mean (range)	NA	9.18 (NA) $^{NA, NA}$
	n	0	1
12	Mean (range)	1.70 (1.70–1.70)	NA
	n	6	0

For analyses and summaries, samples in which infectious virus was detected at levels > ULOQ were assigned a value of 9.18 log_{10} pfu/mL, and samples in which infectious virus was not detected were assigned a value of 1.70 log_{10} pfu/mL. Statistical significance from within- and between-group comparisons, respectively (see Statistical Analysis above), is noted in superscript next to the serum infectious virus values. The significance noted is as follows: ns (not significant) = $p > 0.05$, *$p \leq 0.05$, **$p \leq 0.01$, ***$p \leq 0.001$, ****$p \leq 0.0001$. NA = not applicable.

Supplemental Telemetry Figures

Figure S1: Running Plot of the 30-min Average Body Temperature Analysis for Individual Subjects

Analysis of body temperature (°C). Body temperature values displaying normal (♦), fever (♦), hyperpyrexia (♦), and hypothermia (♦); baseline average values are in gray (▬). Anesthesia use (▲).

***Figure S1**, Panels A–F: Mock-Exposed*

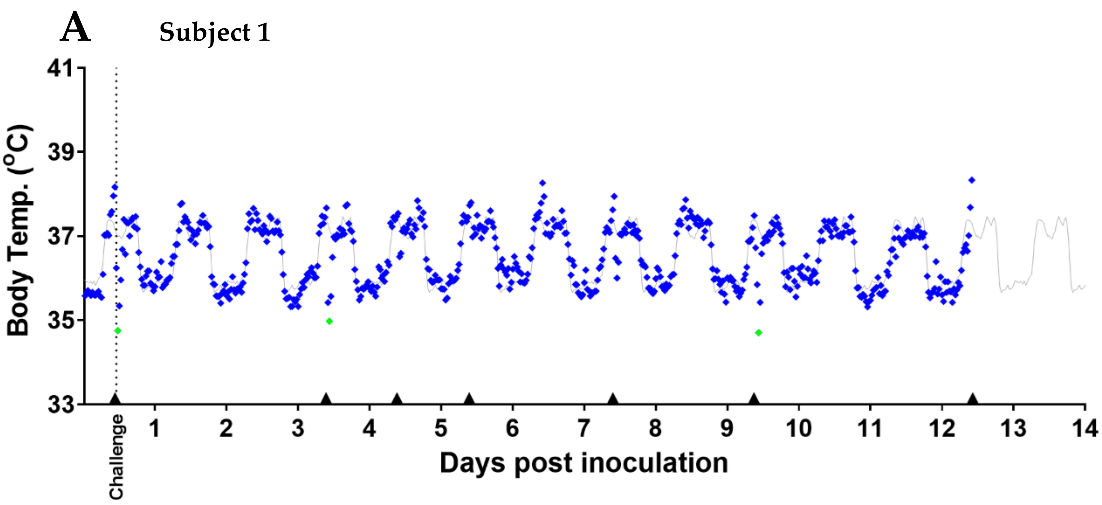

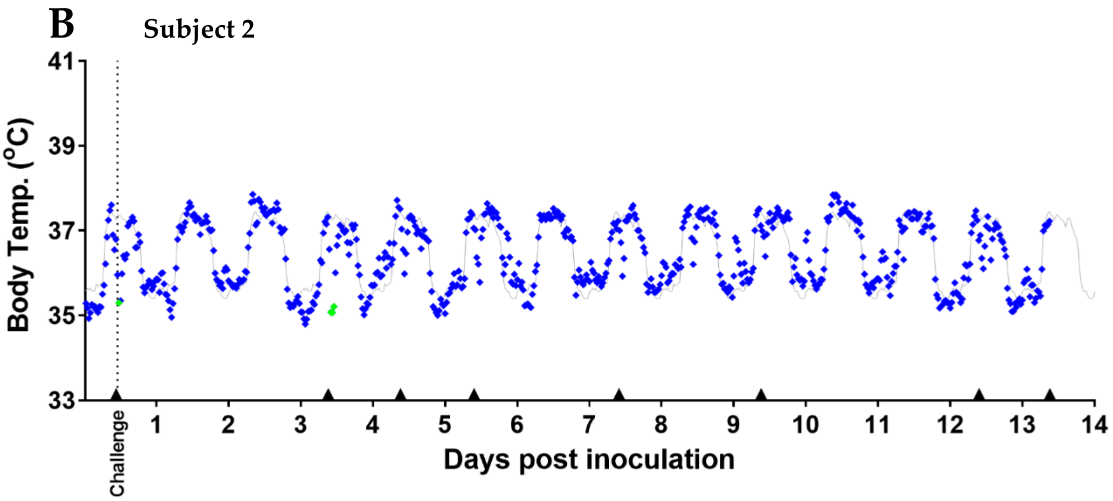

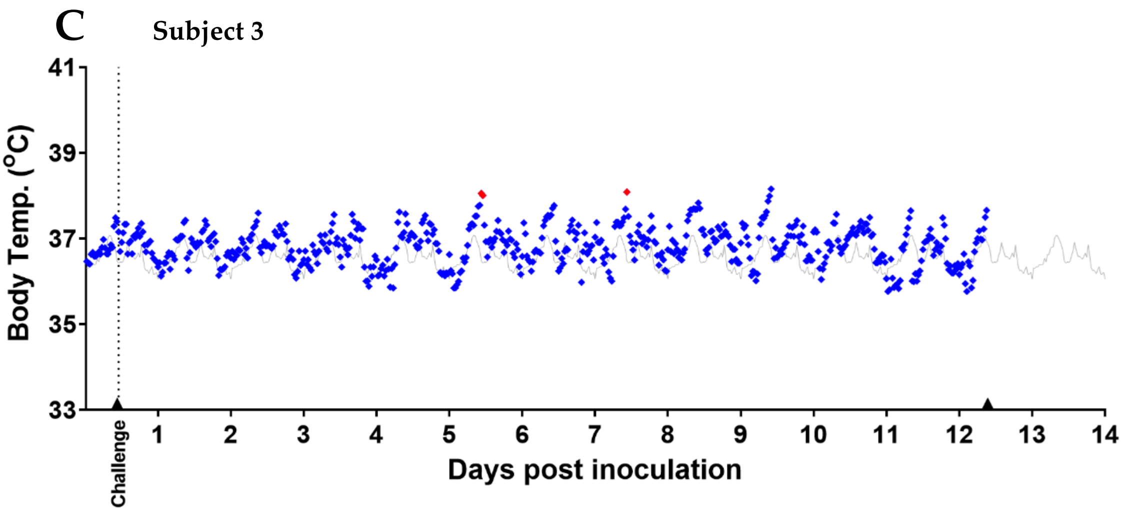
C
Subject 3
Body Temp. (°C)
41
39
37
35
33
Challenge
1
2
3
4
5
6
7
8
9
10
11
12
13
14
Days post inoculation

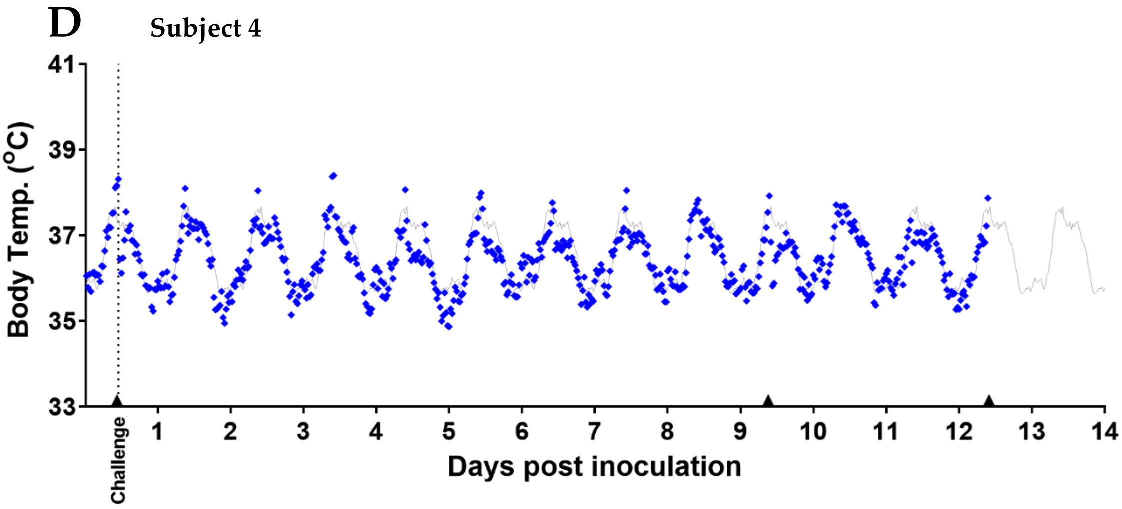
D
Subject 4
Body Temp. (°C)
41
39
37
35
33
Challenge
1
2
3
4
5
6
7
8
9
10
11
12
13
14
Days post inoculation

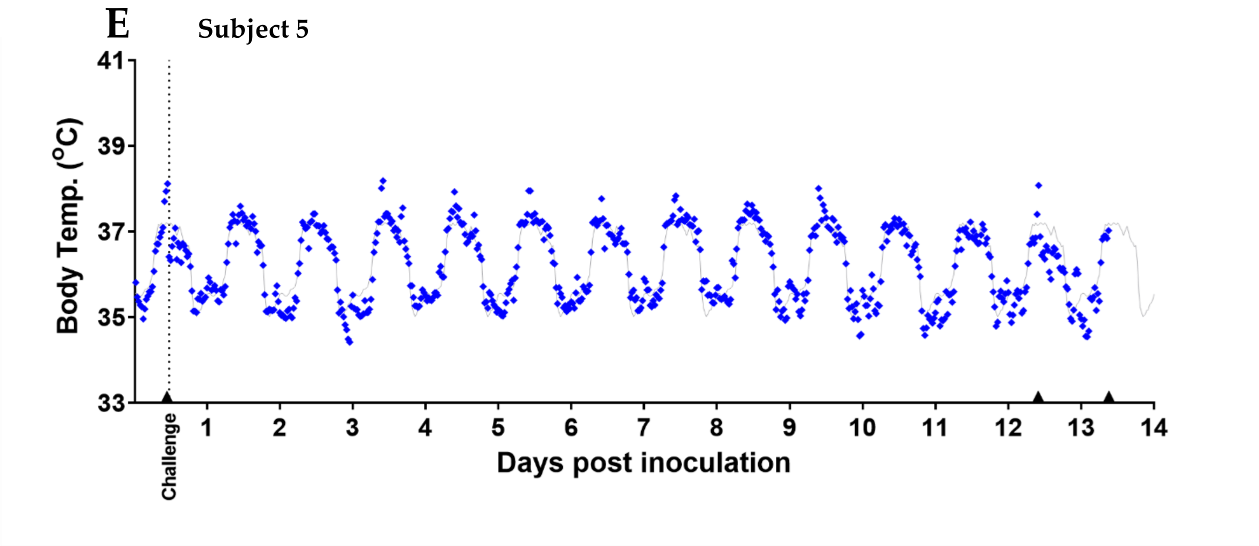
E
Subject 5
Body Temp. (°C)
41
39
37
35
33
Challenge
1 2 3 4 5 6 7 8 9 10 11 12 13 14
Days post inoculation

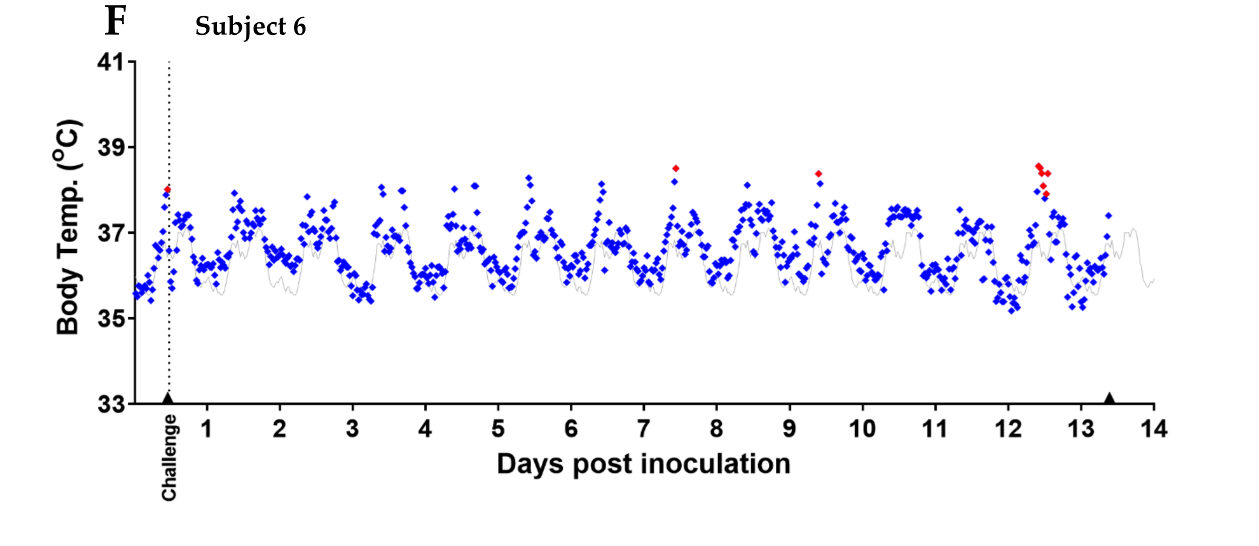
F
Subject 6
Body Temp. (°C)
41
39
37
35
33
Challenge
1 2 3 4 5 6 7 8 9 10 11 12 13 14
Days post inoculation

Figure S1, *Panels G–R: MARV-Exposed*

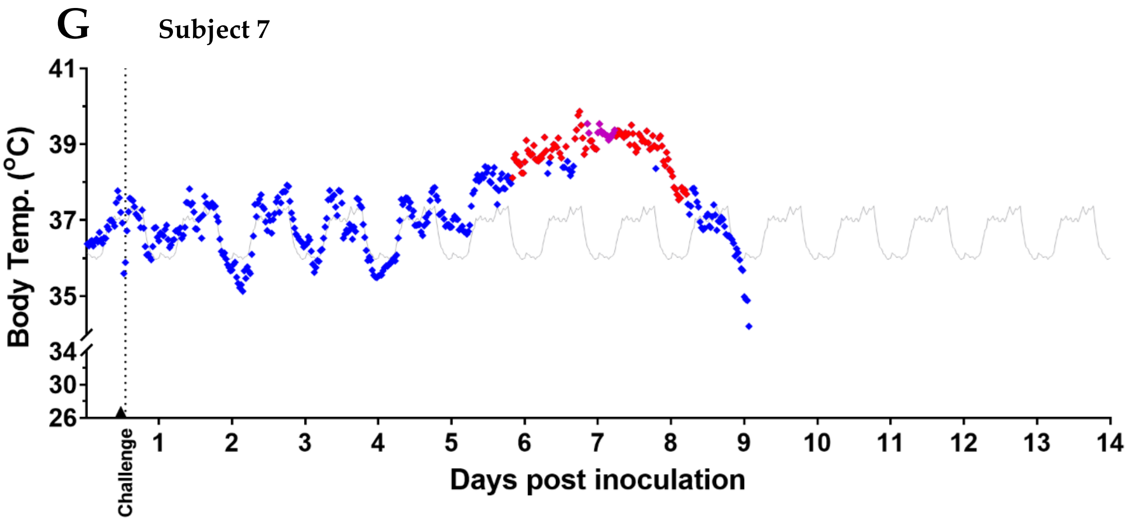

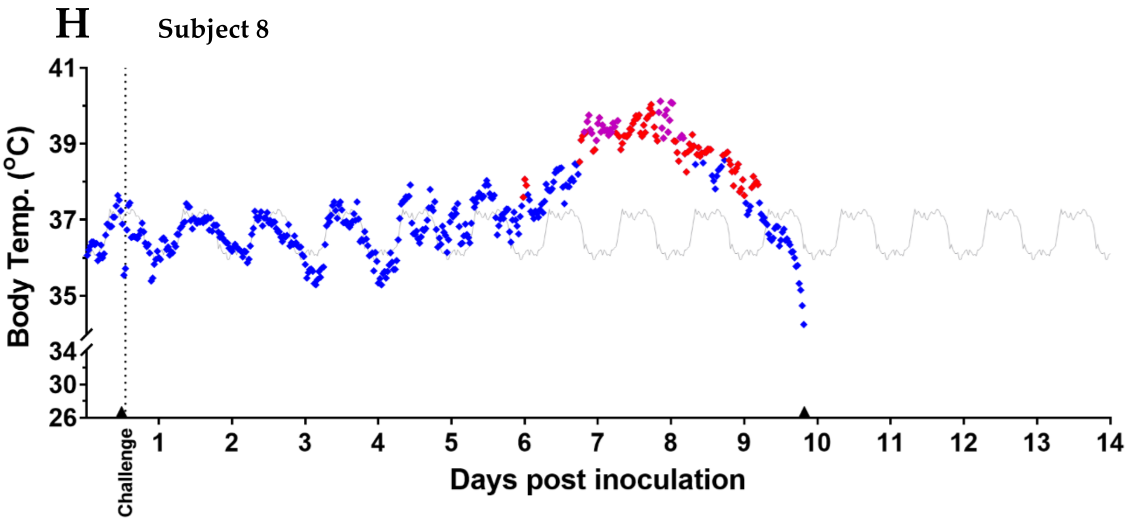

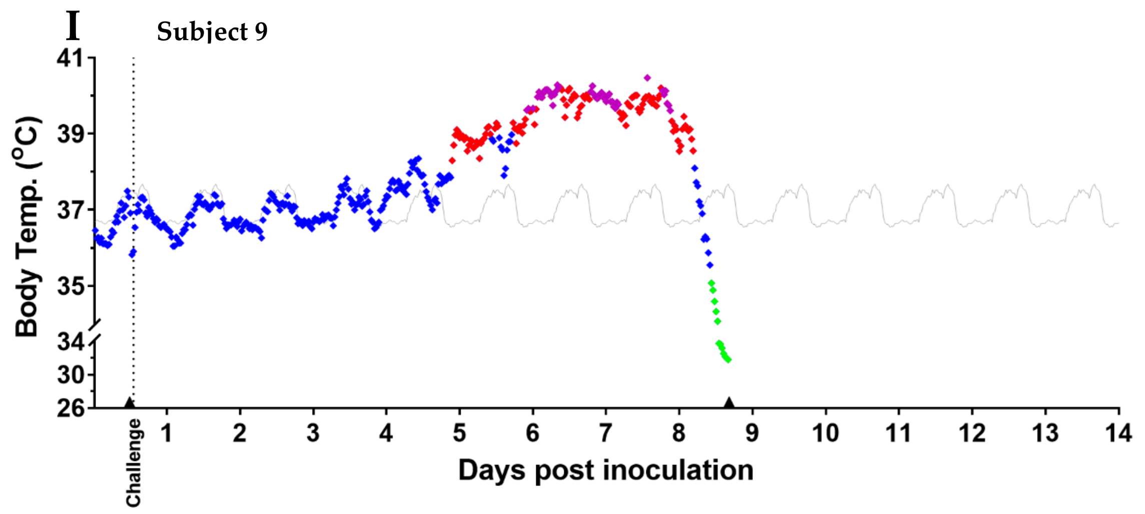
I
Subject 9
Body Temp. (°C)
41
39
37
35
34
30
26
Challenge
1 2 3 4 5 6 7 8 9 10 11 12 13 14
Days post inoculation

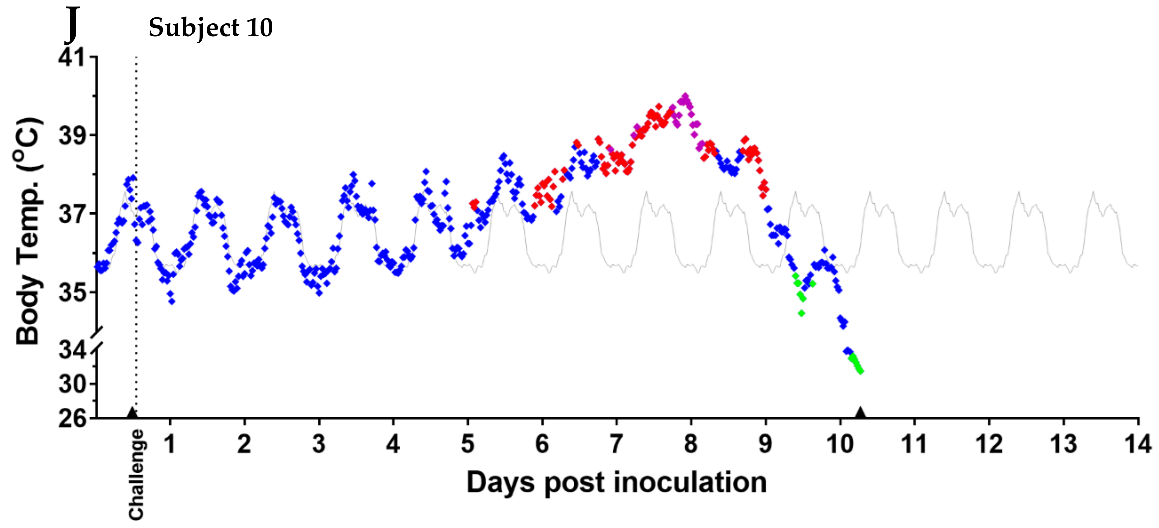
J
Subject 10
Body Temp. (°C)
41
39
37
35
34
30
26
Challenge
1 2 3 4 5 6 7 8 9 10 11 12 13 14
Days post inoculation

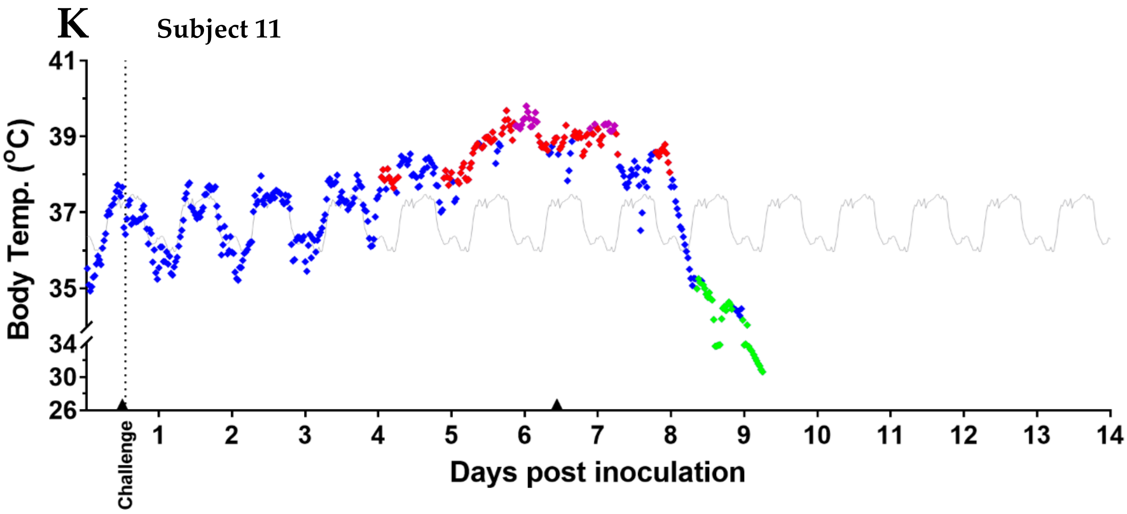
K
Subject 11
Body Temp. (°C)
41
39
37
35
34
30
26
Challenge
1 2 3 4 5 6 7 8 9 10 11 12 13 14
Days post inoculation

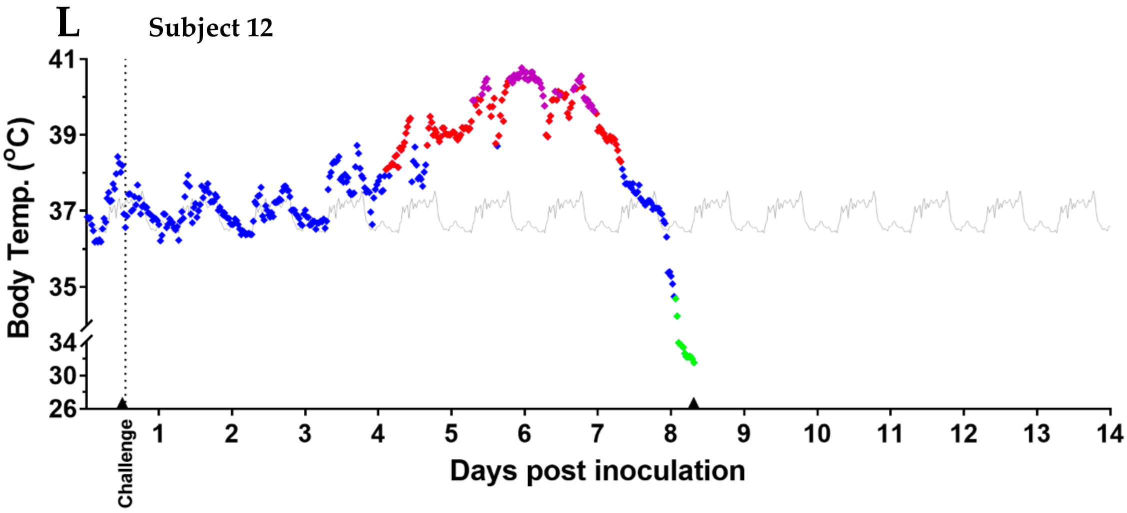
L
Subject 12
Body Temp. (°C)
41
39
37
35
34
30
26
Challenge
1 2 3 4 5 6 7 8 9 10 11 12 13 14
Days post inoculation

M **Subject 13**

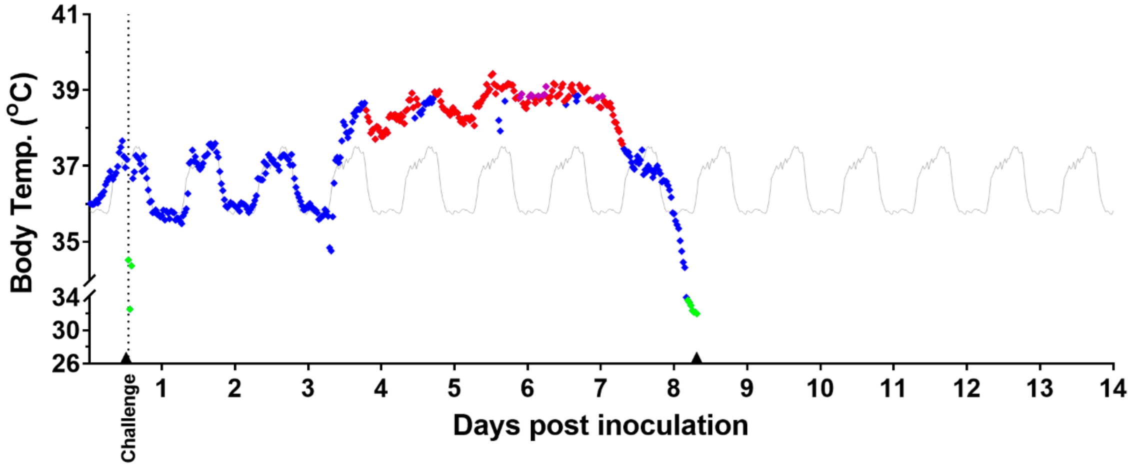

N **Subject 14**

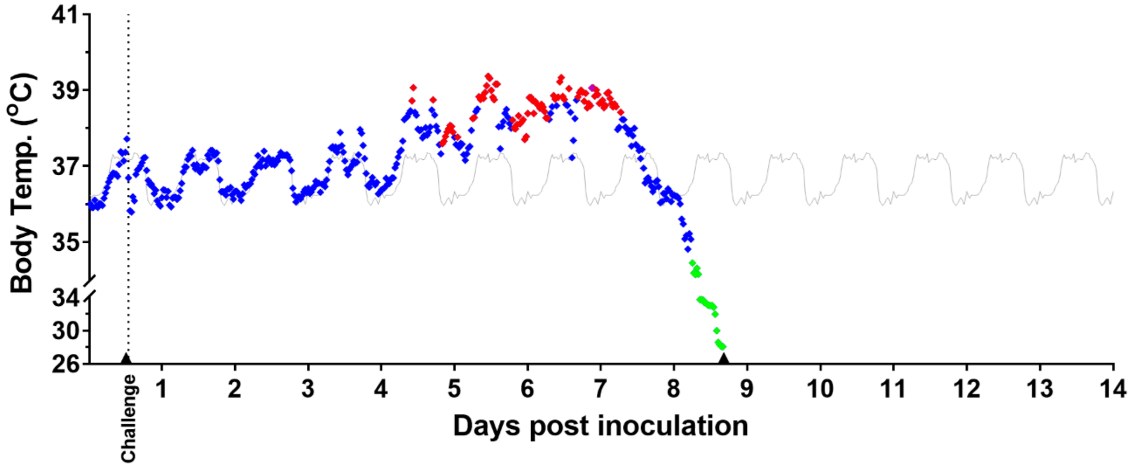

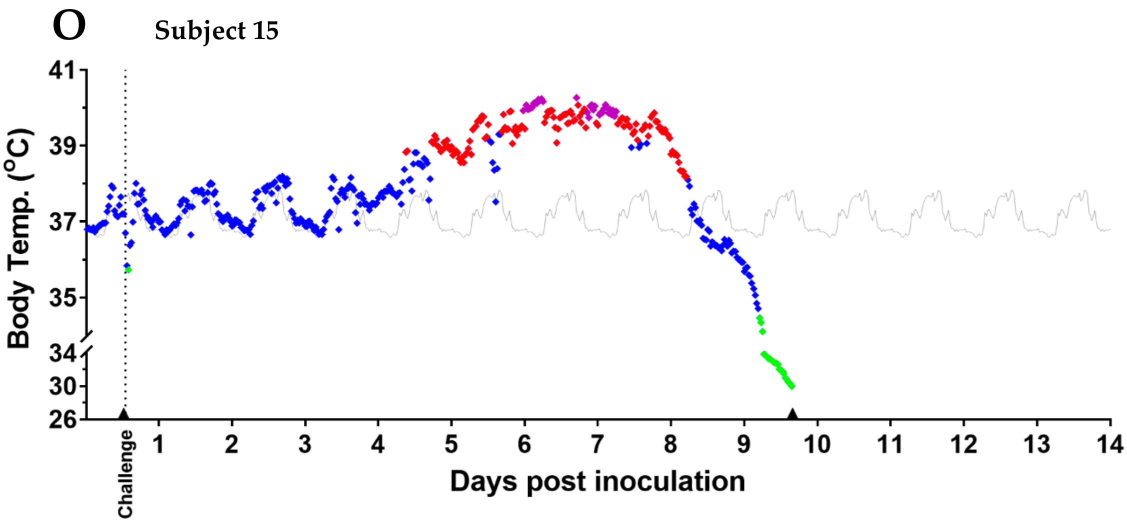
O
Subject 15
Body Temp. (°C)
41
39
37
35
34
30
26
Challenge
1 2 3 4 5 6 7 8 9 10 11 12 13 14
Days post inoculation

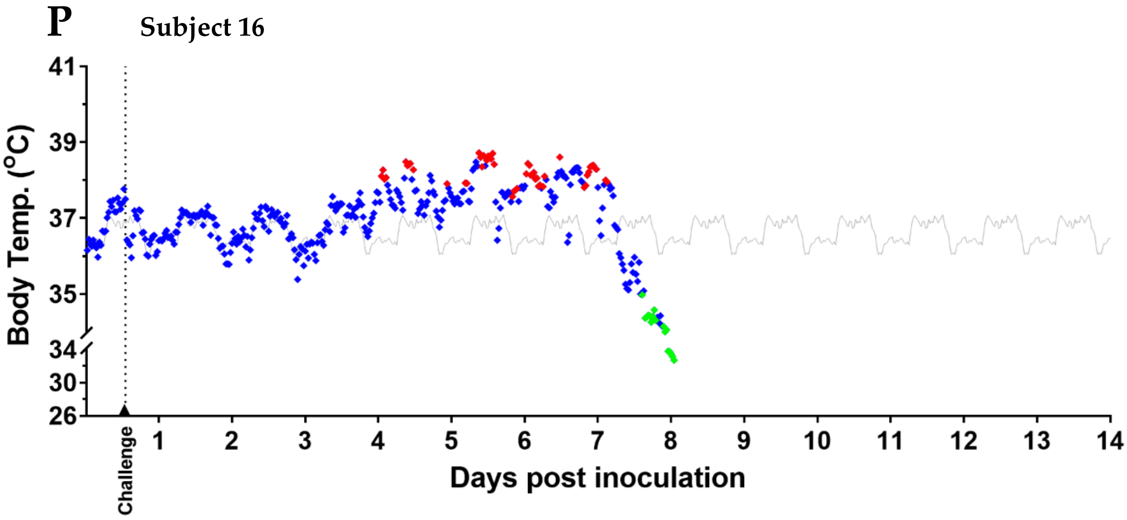
P
Subject 16
Body Temp. (°C)
41
39
37
35
34
30
26
Challenge
1 2 3 4 5 6 7 8 9 10 11 12 13 14
Days post inoculation

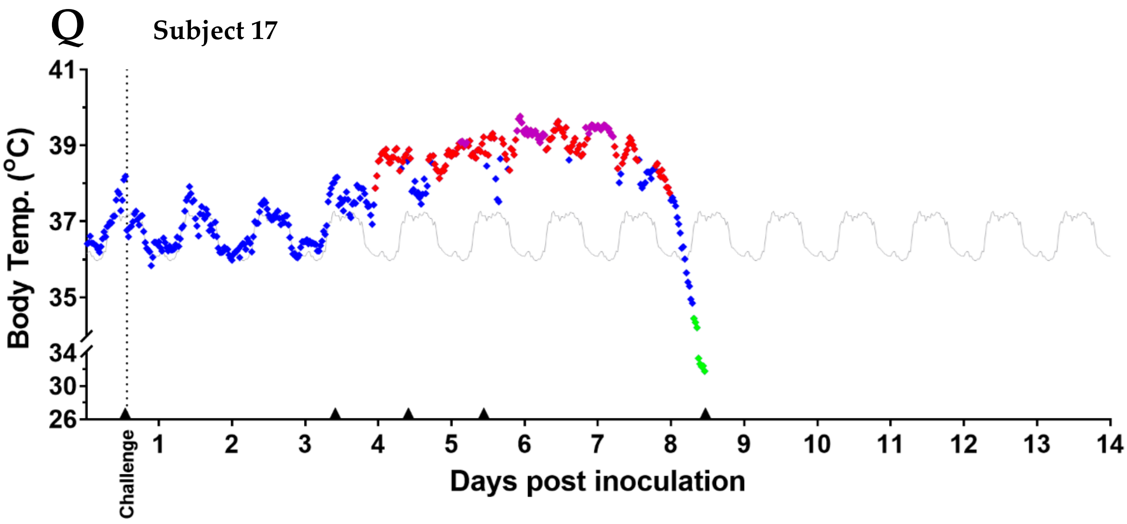
Q
Subject 17
Body Temp. (°C)
41
39
37
35
34
30
26
Challenge
1
2
3
4
5
6
7
8
9
10
11
12
13
14
Days post inoculation

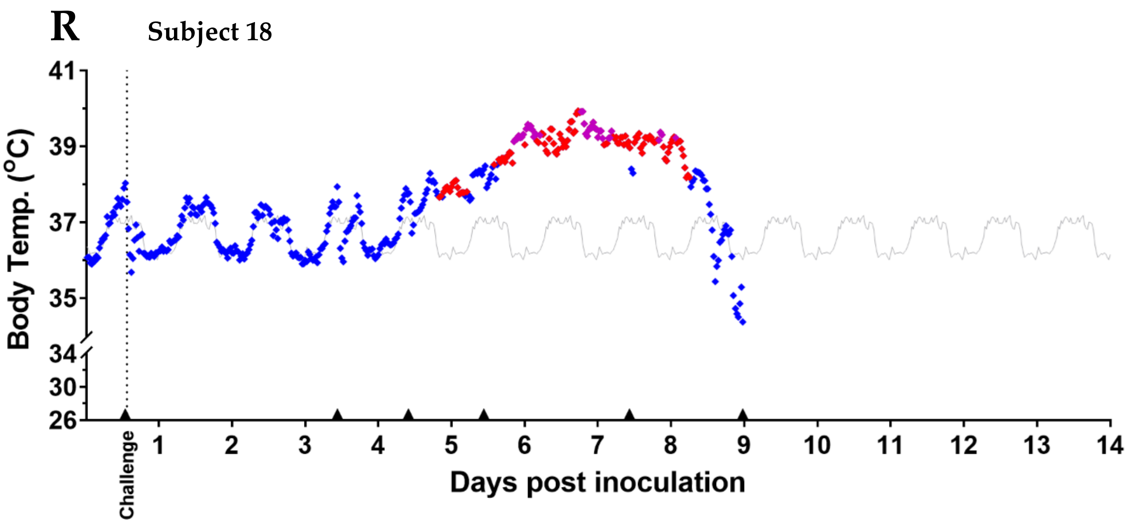
R
Subject 18
Body Temp. (°C)
41
39
37
35
34
30
26
Challenge
1
2
3
4
5
6
7
8
9
10
11
12
13
14
Days post inoculation

Figure S2: 12-hr Average Activity Analysis

Values +3 SD (♦) or -3 SD (♦) from baseline are statistically significant; values < 3 SD (♦) are not significant. Daytime (0600–1800 hours) values are shown with solid blue lines (—♦—); nighttime (1800–0600 hours) values are shown with dotted blue lines (- - -♦- - -). Baseline average values for daytime and nighttime are shown as black dotted lines (- - - -).

Figure S2*, Panels A–F: Mock-Exposed*

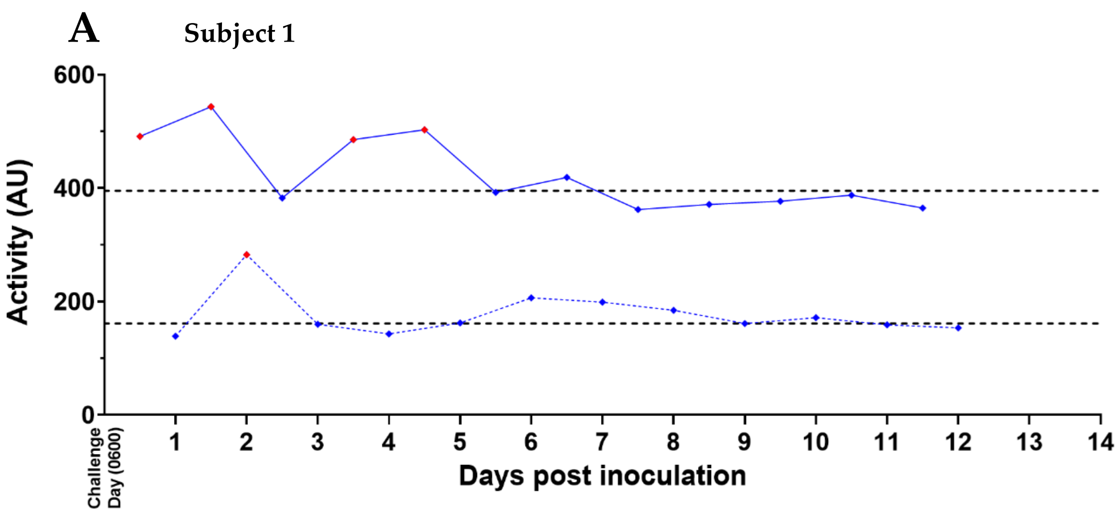

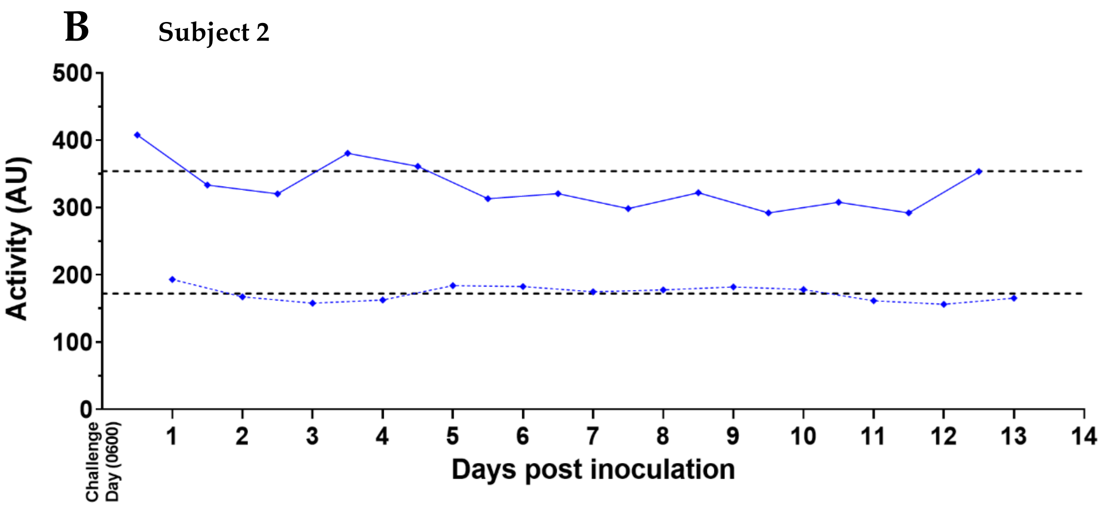

C **Subject 3**

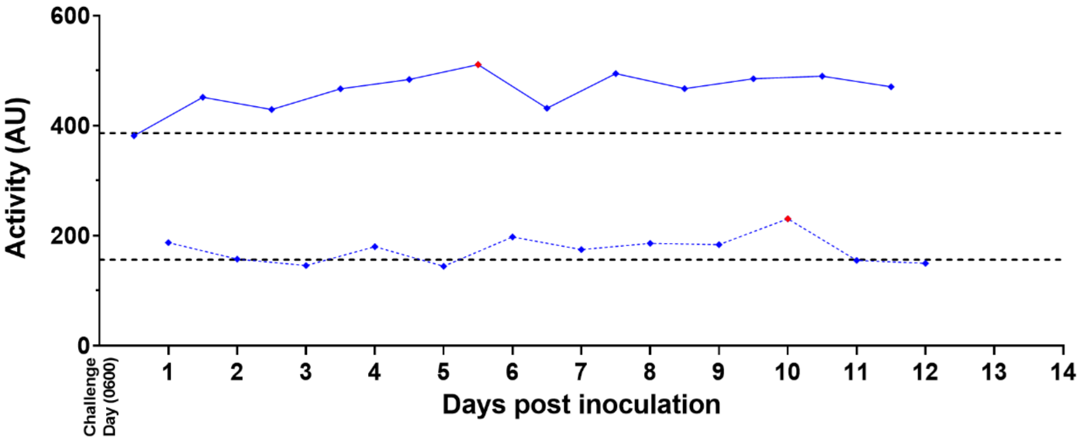

D **Subject 4**

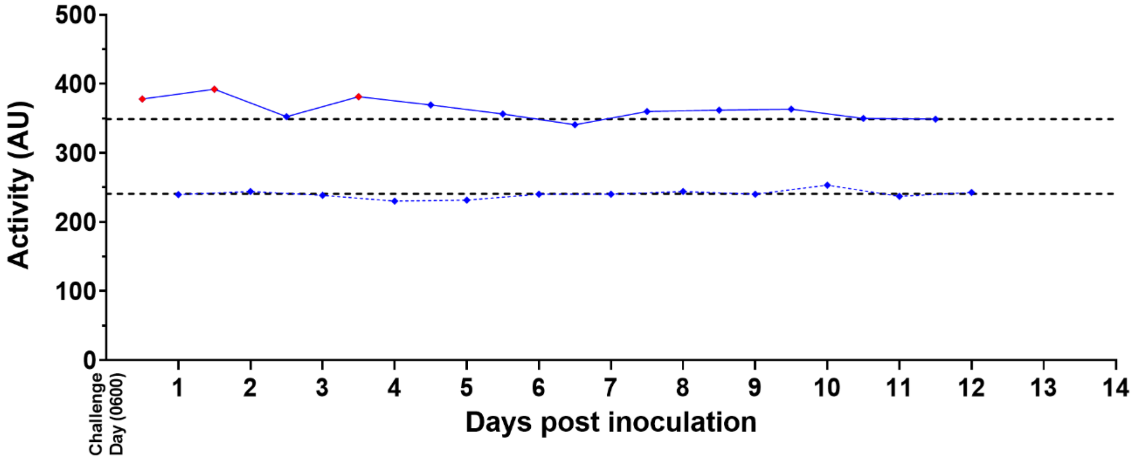

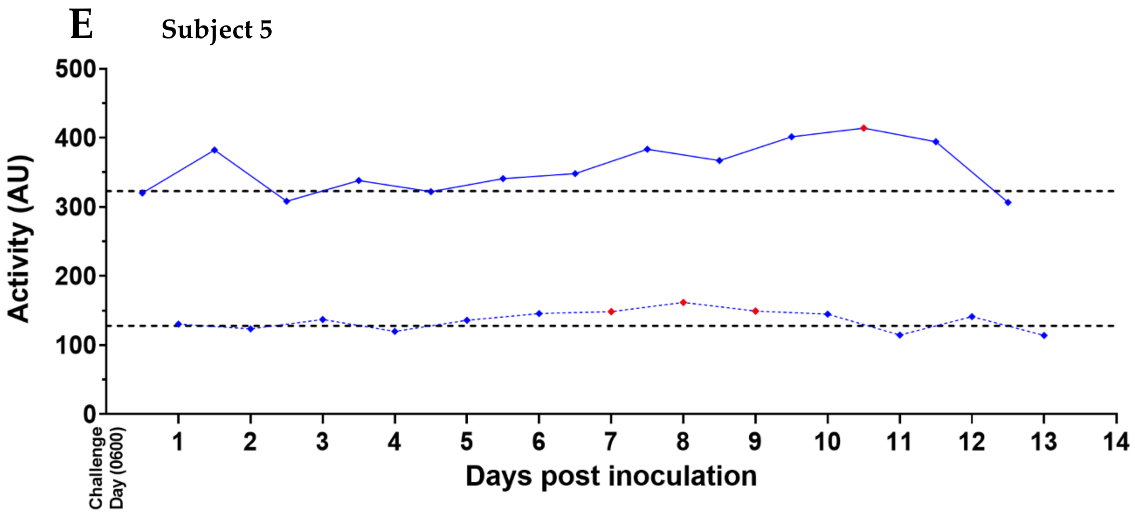
E
Subject 5
Activity (AU)
500
400
300
200
100
0
Challenge Day (0600)
1
2
3
4
5
6
7
8
9
10
11
12
13
14
Days post inoculation

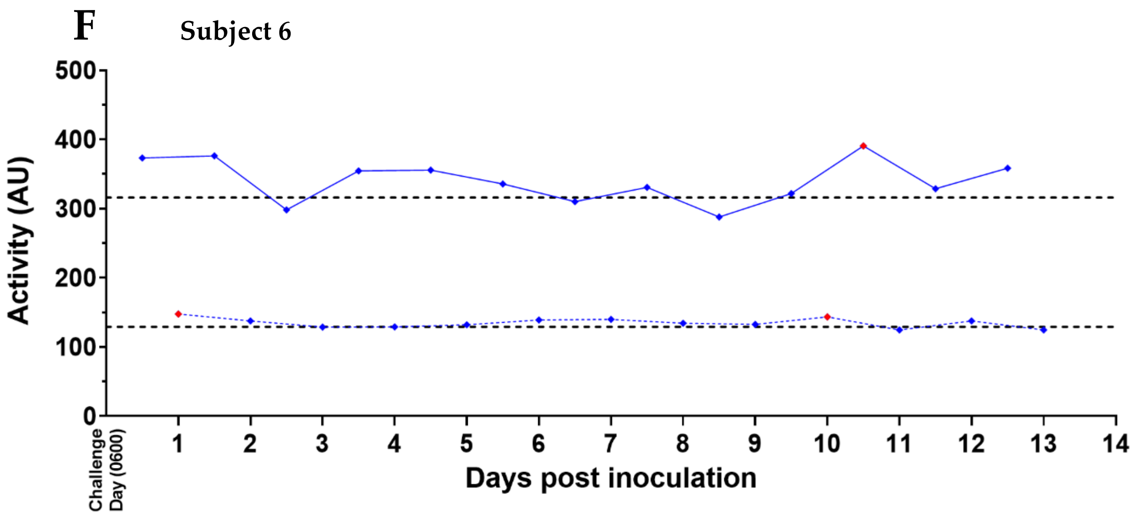
F
Subject 6
Activity (AU)
500
400
300
200
100
0
Challenge Day (0600)
1
2
3
4
5
6
7
8
9
10
11
12
13
14
Days post inoculation

Figure S2, *Panels G–R: MARV-Exposed*

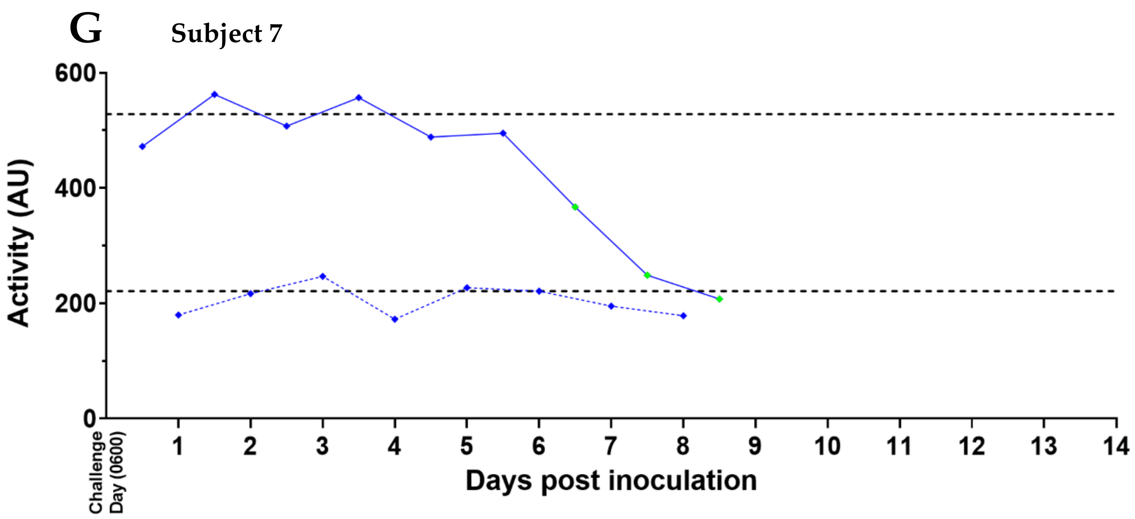

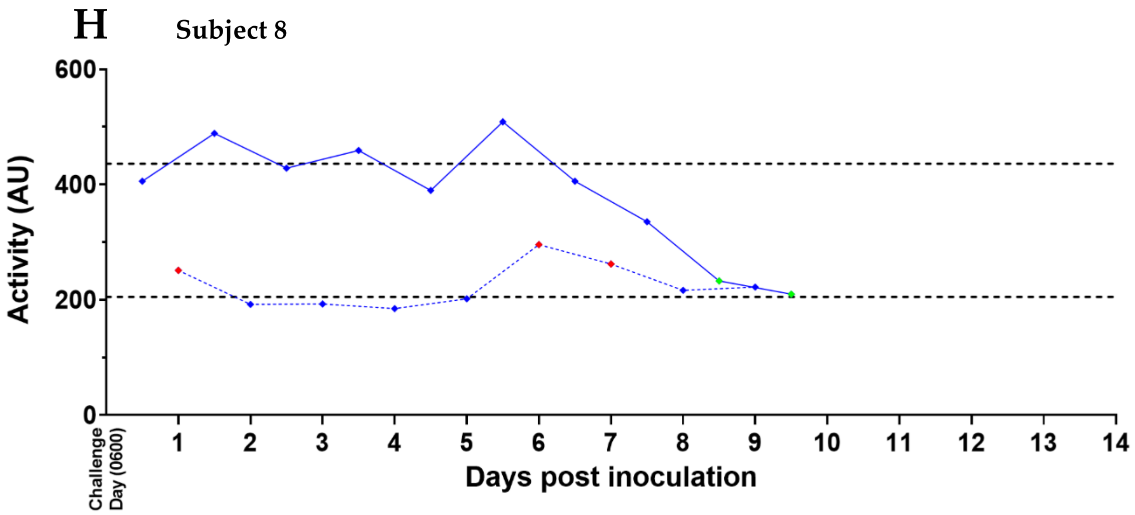

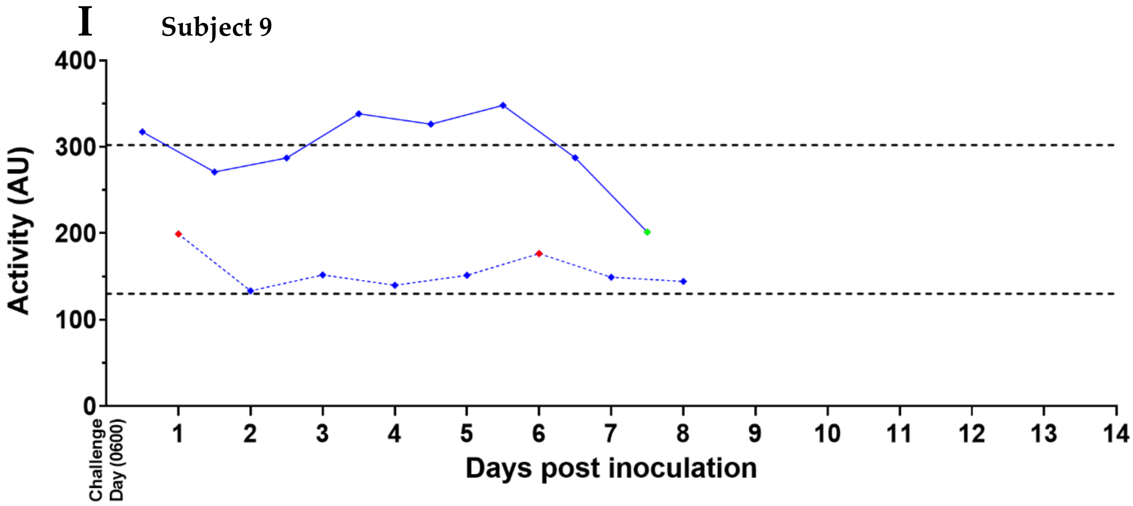
I
Subject 9
400
300
200
100
0
Activity (AU)
Challenge Day (0600)
1 2 3 4 5 6 7 8 9 10 11 12 13 14
Days post inoculation

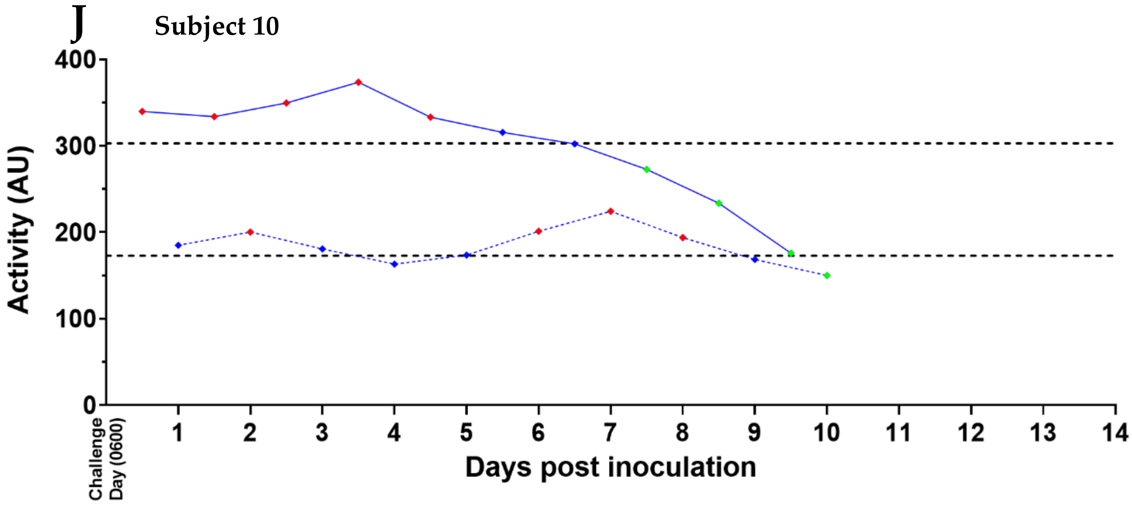
J
Subject 10
400
300
200
100
0
Activity (AU)
Challenge Day (0600)
1 2 3 4 5 6 7 8 9 10 11 12 13 14
Days post inoculation

K **Subject 11**

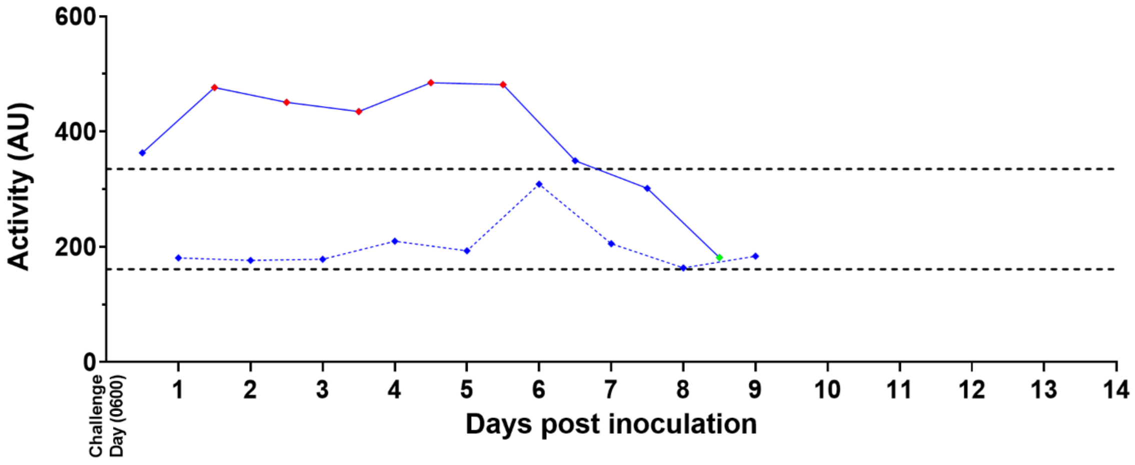

L **Subject 12**

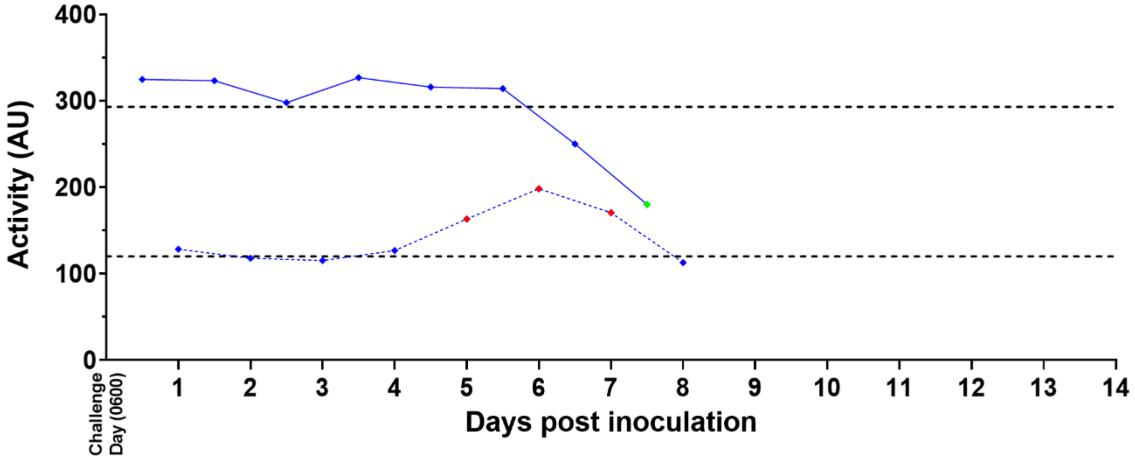

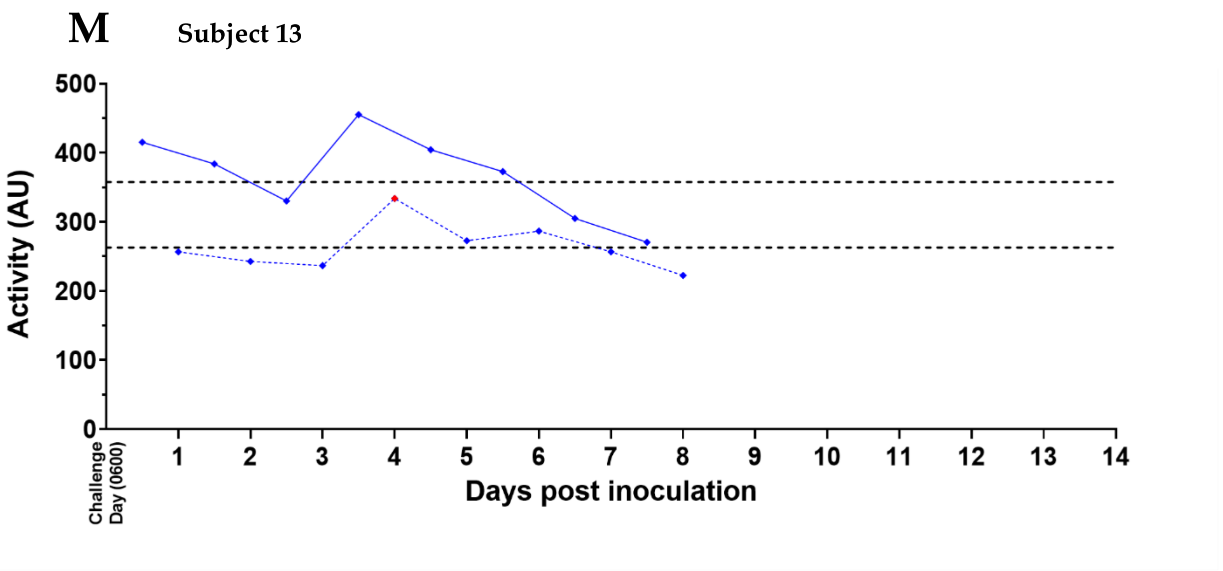
M
Subject 13
500
400
300
200
100
0
Activity (AU)
Challenge Day (0600)
1
2
3
4
5
6
7
8
9
10
11
12
13
14
Days post inoculation

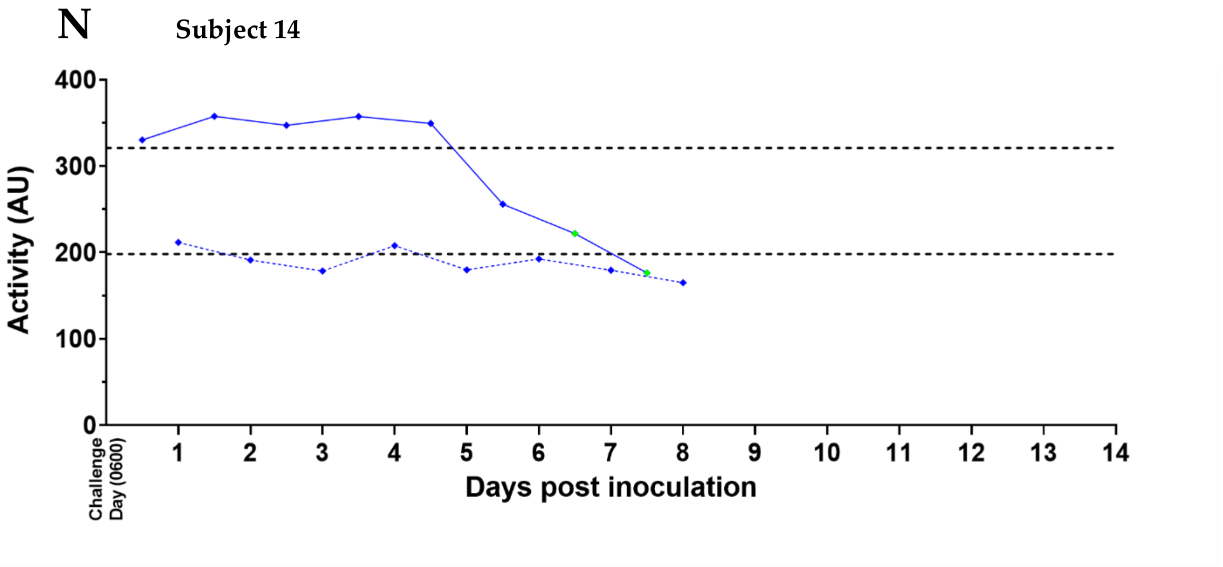
N
Subject 14
400
300
200
100
0
Activity (AU)
Challenge Day (0600)
1
2
3
4
5
6
7
8
9
10
11
12
13
14
Days post inoculation

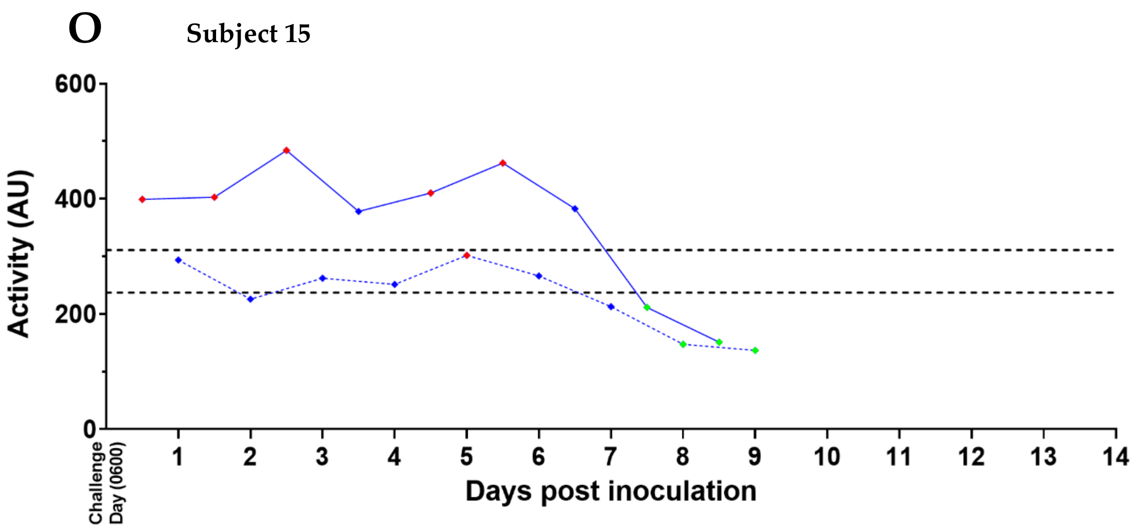
O
Subject 15
600
400
200
0
Activity (AU)
Challenge Day (0600)
1
2
3
4
5
6
7
8
9
10
11
12
13
14
Days post inoculation

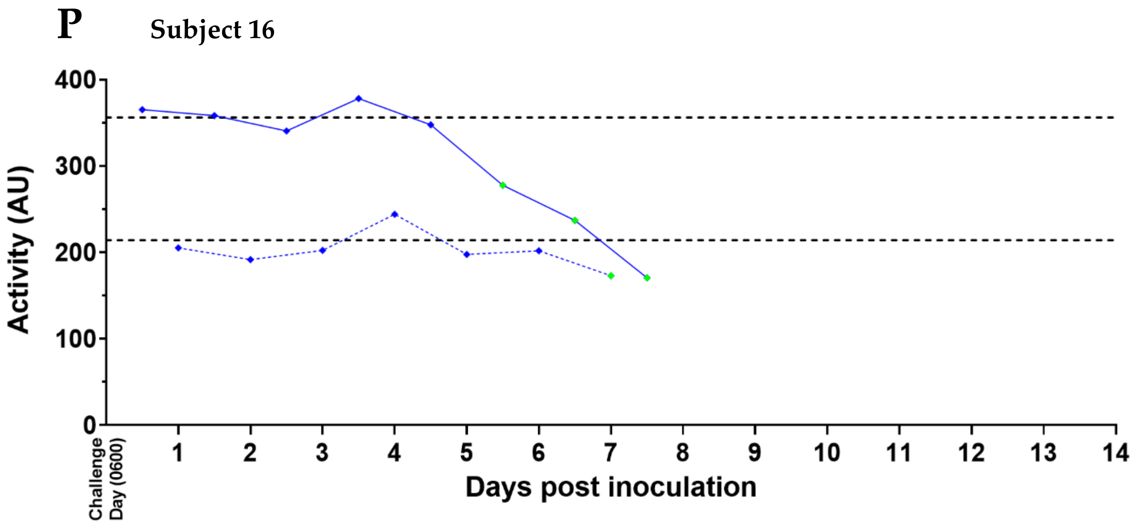
P
Subject 16
400
300
200
100
0
Activity (AU)
Challenge Day (0600)
1
2
3
4
5
6
7
8
9
10
11
12
13
14
Days post inoculation

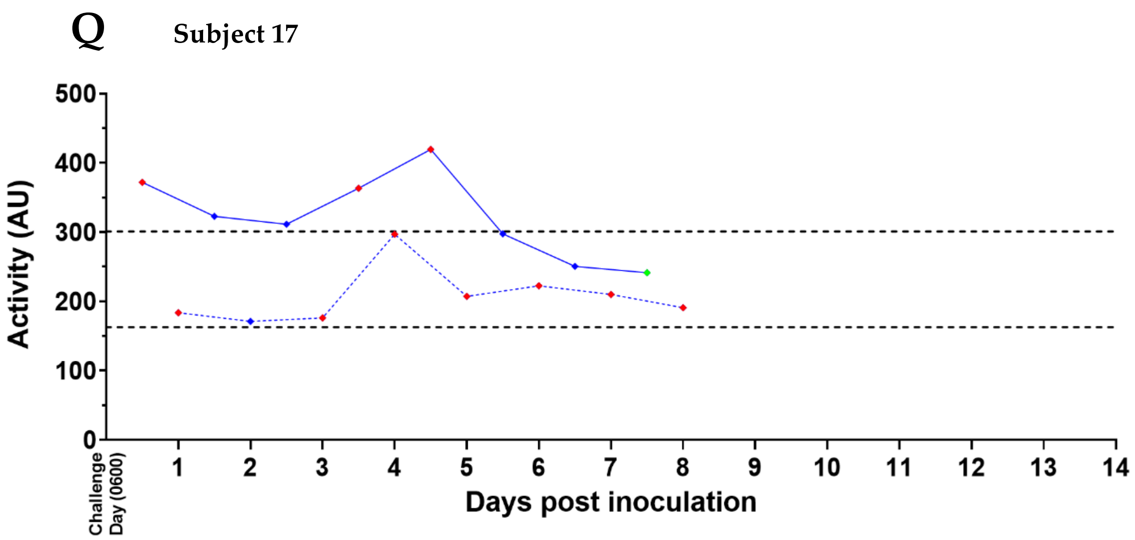
Q
Subject 17
Activity (AU)
500
400
300
200
100
0
Challenge Day (0600)
1 2 3 4 5 6 7 8 9 10 11 12 13 14
Days post inoculation

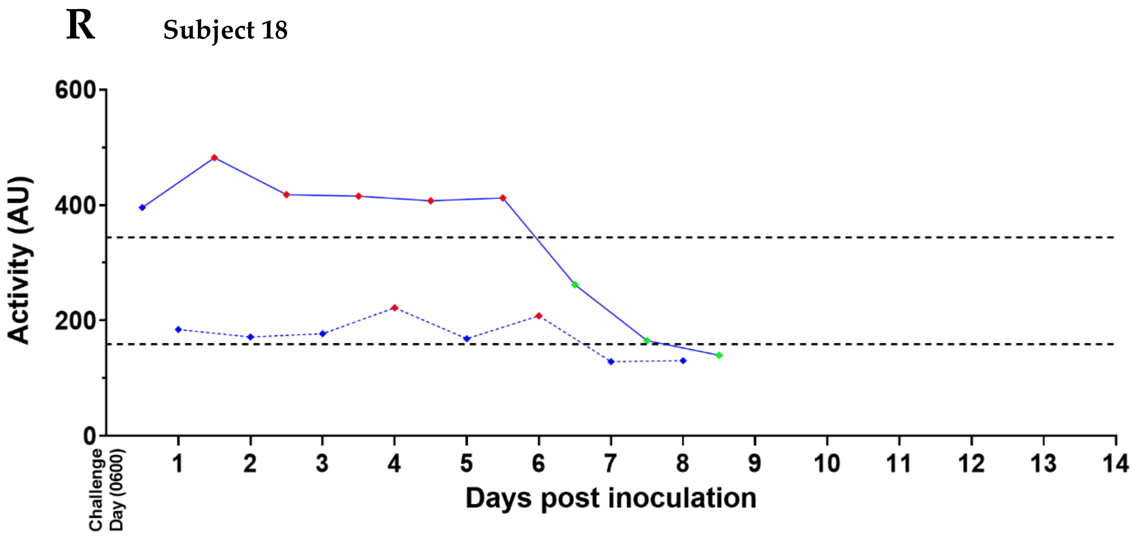
R
Subject 18
Activity (AU)
600
400
200
0
Challenge Day (0600)
1 2 3 4 5 6 7 8 9 10 11 12 13 14
Days post inoculation
